# Clinical improvement and radiological progression in a girl with early onset scoliosis (EOS) treated conservatively – a case report

**DOI:** 10.1186/1748-7161-1-13

**Published:** 2006-07-26

**Authors:** Hans-Rudolf Weiss

**Affiliations:** 1 Asklepios Katharina Schroth Spinal Deformities Rehabilitation Centre, Bad Sobernheim, Germany

## Abstract

**Background:**

Chêneau-Brace treatment of a certain standard reduces the rate of surgery, prevents progression and in a certain patient population leads to marked improvement of Cobb angle and cosmetic appearance. During the last two years a patient refusing surgery with a double major curvature of initially 60° showed a clear cosmetic improvement and a clear radiological progression at the same time. The findings of this patient have been reviewed in order to find out how cosmetic appearance and Cobb angle can develop differently.

**Methods:**

The patient entered conservative treatment at the age of 13 years, premenarchial with Tanner II and a Cobb angle of 60° thoracic and 59° lumbar. The angle of trunk rotation (ATR; Scoliometer) was 13° thoracic and 13° lumbar. We have documented the findings of this patient (Surface topography, ATR, Cobb angles and angles of vertebral rotation (according to Raimondi) during the treatment period (27 Month) until 2 years after the onset of menarche.

**Results:**

After a treatment time of 27 Month the Cobb angle increased to 74° thoracic and 65° lumbar. The angles of vertebral rotation according to Raimondi increased slightly from 26° thoracic and 28° lumbar to 30° thoracic and 28° lumbar. The ATR improved to 12° thoracic and 5° lumbar while Lateral deviation improved from 22,4 mm to 4,6 mm and average surface rotation improved from 10,6° to 6°. In the X-rays a reduction of decompensation was visible. The patient felt comfortable with the cosmetic result.

**Conclusion:**

Conservative treatment may improve cosmetic appearance while the curve progresses radiologically. This could be explained by assuming that (1) the Rigo Chêneau brace is able to improve cosmetic appearance by changing the shape of the thorax when the curve itself is too stiff to be corrected by a brace, that (2) reduction of decompensation leads to significant cosmetical improvements or (3) that the patient gained weight and therefore the deformation is masked. However, the weight the patient gained cannot explain the cosmetical improvement in this case.

Conservative treatment with a certain standard of quality seems a viable alternative for patients with Cobb angles of > 60° when surgical treatment is refused.

Specialists in scoliosis management should be aware of the fact that curve progression can occur even if the clinical measurements show an improvement.

## Background

In Continental Europe [1-3] especially in Germany, a conservative treatment approach is pursued actively from the time of diagnosis [4-9]. In adolescence, this approach includes outpatient physiotherapy beginning at 15° according to Cobb. Scoliosis intensive rehabilitation (SIR) is recommended for curvatures of 20° to 30°, with or without bracing, depending on prognosis [10]. For adult Idiopathic Scoliosis (IS), outpatient physiotherapy is offered for curvatures of 30° to 40° [11-13] with moderate pain. Physiotherapists in different regions are trained, so that patients have the option of continued outpatient treatment close to their residence. For adult patients with curves over 40° in association with cardio-respiratory functional impairment and pain, SIR is recommended. In-patient treatment offers structure for a daily six-hour intensive rehabilitation treatment [11].

Two factors have emerged as the main parameters of successful brace treatment. Goldberg and co-workers [14] cite two references in which good patient compliance with bracing corresponded with favorable outcomes [15,16]. However, the actual extent of the corrective effect is also described as an essential criterion in successful bracing. Based on a review of the literature, we confirmed that there exists a direct positive correlation between the primary corrective effect of an orthosis and the end result [17]. The importance of this effect is supported by a study from Mellerowicz et. al. [18] and by a study from Landauer [19], in which they independently conclude that compliance and the primary correction effect in the brace are the two most important variables associated with good brace outcomes.

**Figure 1:**
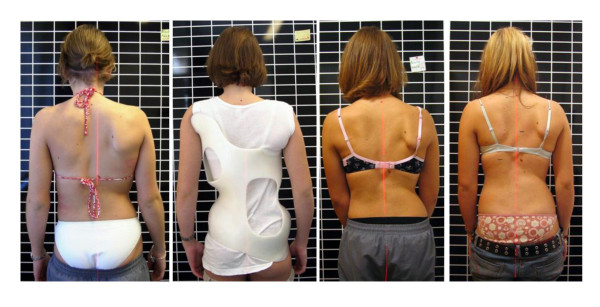
**Clinical history of the patient**. On the left the patient can be seen at the start of treatment, on the middle left in the first brace, on the middle right clinically after one year of treatment and on the right after 27 months of treatment before the weaning brace was adjusted. A considerable cosmetic improvement can be seen comparing the picture at the start of treatment (left) to the picture two years after the onset of menarche (right).

**Figure 2:**
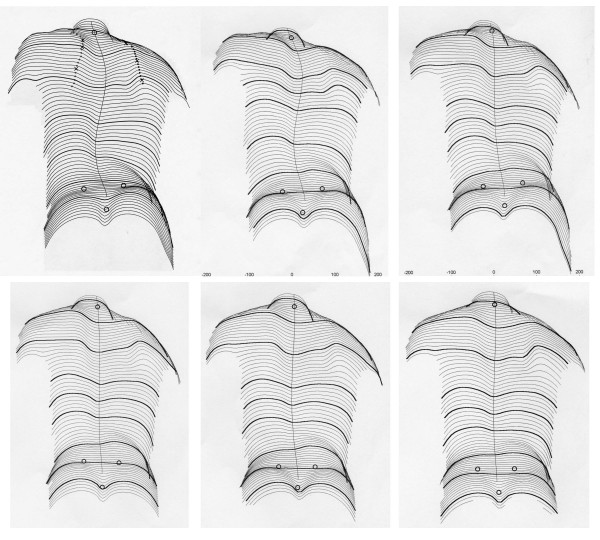
**Clinical history of the patient as documented by surface topography**. On the upper left the patients reconstructed back can be seen at the start of treatment, on the upper middle picture 6 months and on the upper right picture 12 months while treated with the brace. A little improvement is visible after 12 month of treatment. On the lower left the patients reconstructed back can be seen 15 month after the start of treatment, on the lower middle picture 20 months and on the lower right picture 27 months while treated. A clear cosmetic improvement is visible when the initial surface reconstruction (upper left) is compared to the last one on the lower right.

The treatment of AIS however serves to change not only the secondary symptoms of scoliosis and the x-ray (Fig. 1 and 2) but – most important to the adolescent – also aims at an improvement of the cosmetic signs of the deformity [10,14]. Thulbourne and Gillespie [20] may be right in saying that even if the progression can be reduced by bracing, cosmetic appearance and the rib hump may not always be influenced positively, nor may a successful course as shown by X-ray always be appreciated as a successful treatment by the patient. The authors express their point of view as follows: *Ever since the introduction of the Milwaukee brace, opinions have differed widely as to its effect on the rib hump. We are now able to state that in most cases the true rib hump on the convex side of a thoracic curve is little influenced, whereas the contralateral rib depression can be markedly reduced, and in the most responsive cases even completely corrected. Thus for patients where the rib depression contributes a disproportionate part of the total deformity, one may maintain a rather more optimistic view as to the ultimate clinical appearance than can be held when the hump is the more obvious feature *[20].

Rigo however has demonstrated that the application of the Rigo-System Chêneau brace (RSC-Brace) leads to significant improvements of the cosmetic deformations [21,22] and to a significantly reduced rate of progression [23]. The frontal corrections in the RSC brace were at average about 30 – 40% for the major curve but also showed a significant reduction of vertebral rotation (22%). So not only compliance and correction in frontal plane but also 3-D correction plays an important role in the conservative management with the help of braces.

Treatment of scoliosis with the Chêneau brace is currently the most practised conservative method in Germany, Austria, Spain, Greece as well as in Poland. It is also used in France and Italy. The Cheneau brace is defined as a thermoplastic brace modelled on a hyper-corrected positive plaster mould of the patient. Specific pad areas are designed to provide detorsional forces through the trunk. Expansion rooms are also provided, in order to allow active correction by breathing movements. Clinical histories of individuals enjoying excellent corrective effects and favorable outcomes with the Cheneau brace are encouraging its use in many places in Central Europe.

During the last two years a patient refusing surgery with a double major curvature of initially 60° showed a clear cosmetic improvement and a clear progression at the same time while treated conservatively with out-patient exercises, in-patient rehabilitation and a Rigo-System Chêneau brace. The findings of this patient have been reviewed in order to find out how cosmetic appearance and Cobb angle can develop differently.

## Methods

The patient with EOS (onset at the age of 6 years) entered conservative treatment (out-patient exercises, in-patient rehabilitation and the Chêneau brace) at the age of 13 years, premenarchial with Tanner II and a Cobb angle of 60° thoracic and 59° lumbar. The angle of trunk rotation (ATR; Scoliometer) was 13° thoracic and 13° lumbar. We have documented the findings of this patient (Surface topography, ATR, Cobb angles and angles of vertebral rotation according to Raimondi) during the treatment period (27 months) until 2 years after the onset of menarche.

## Results

### Patient history

After a treatment time of 27 months the Cobb angle increased to 74° thoracic and 65° lumbar. The angles of vertebral rotation according to Raimondi increased slightly from 26° thoracic and 28° lumbar to 30° thoracic and 28° lumbar.

The ATR improved to 12° thoracic and 5° lumbar while lateral deviation (as measured by surface topography) improved from 22,4 mm to 4,6 mm and average surface rotation improved from 10,6° to 6°. In the X-ray an improvement of decompensation was visible. No change in curve pattern occurred. The patient felt comfortable with the cosmetic result. She gained 23 of 24 points in the Bad Sobernheim Stress Questionnaire (BSSQ) which means she feels only very little stress with respect to her deformity of the trunk [24], while she was very concerned at her first presentation.

### Cosmetic changes

The cosmetic history of the patient is documented on figure 1 and 2.

### Radiological course

**Figure 3:**
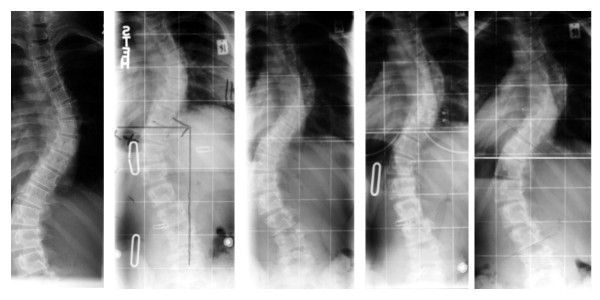
**Radiological history of the patient**. From left (x-ray taken outside our centre before start of treatment) to right the radiological course of the patient can be seen (last picture after 27 months on the right). A progression of the curve is visible.

The radiologic history of the patient is documented on figure 3.

**Figure 4:**
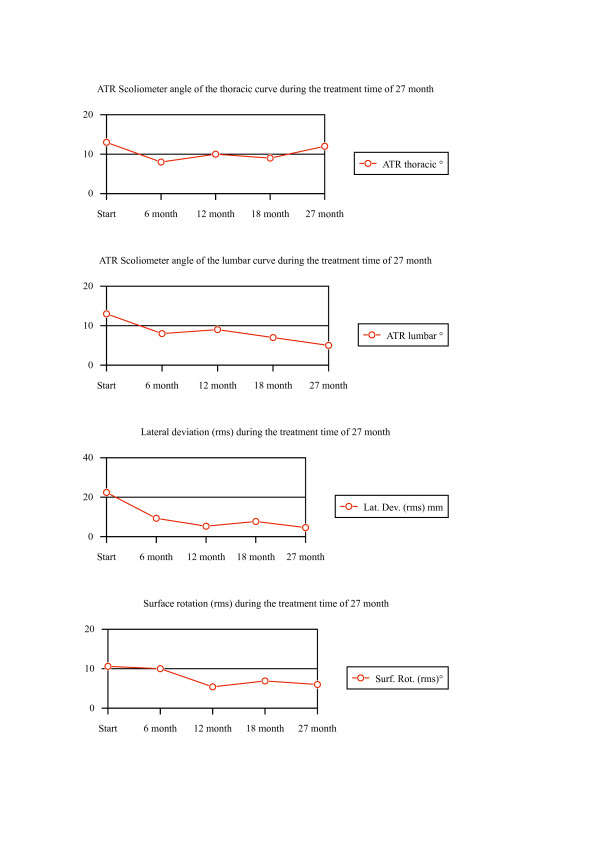
**Clinical history of the patient as documented by angle of trunk rotation (Scoliometer) and surface topography (lateral deviation and surface rotation)**. On the top course of the thoracic ATR and lumbar ATR; on the bottom the course of lateral deviation and surface rotation during the time of treatment (27 Months).

The values describing the cosmetic changes can be seen in Additional file 1 and the history of those values are content of figure 4.

## Discussion

Long-term controlled studies indicate that spinal fusion for Adolescent Idiopathic Scoliosis (AIS), the largest of scoliosis populations does not lead to an improvement of lung function [25], to less pain [26], better „General Health” [27] and less degeneration [28]. So the only clear indication for spinal fusion in AIS patients and many other forms of scoliosis is severe deformity related stress [14], when the possible long-term risks of spinal surgery are respected [29].

Therefore the patient described in this case report can decide herself as to whether be treated conservatively or operatively. During clinical follow-up we were quite satisfied with the cosmetic improvements achieved although the initial Cobb angle was very high and after the follow-up x-ray we were quite surprised that the curve was progressive.

The first explanation for the increase of Cobb angle and the reduced rib-hump at the same time was geometry: If a curve is derotated it turns from a sagitto-frontal direction to frontal and therefore must show a bigger angle on the frontal x-ray. In that case, however the angle of rotation of the apical vertebra should improve at the same time. This is why the x-ray films have been re-evaluated and this time we also measured the rotation of the apical vertebra using the Raimondi device [30]. Interestingly the rotation angles showed to have increased as well.

**Figure 5:**
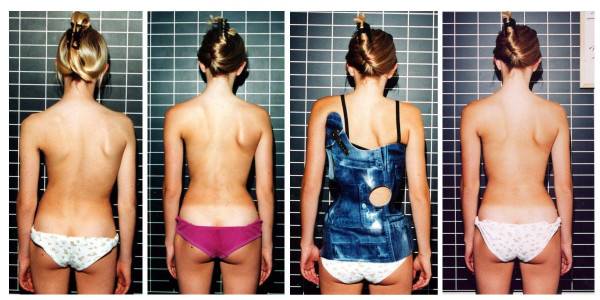
**Clinical history of a patient with 45° at the age of 13 and 27° at the age of 16 after brace weaning**. On the left the patient can be seen at the start of treatment, on the middle left clinically after one year of treatment, on the middle right in her second brace, and on the right after 36 months of treatment after weaning. A considerable cosmetic improvement can be seen comparing the picture at the start of treatment (left) to the picture two years after the onset of menarche (right) while the curve has improved 18° Cobb at the same time.

**Figure 6:**
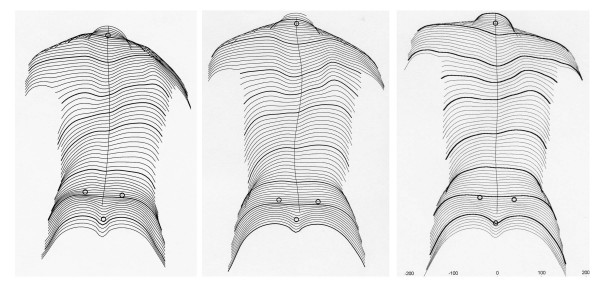
**Clinical history of the patient as documented by surface topography (second year of treatment)**. On the left the patients reconstructed back can be seen at the start of treatment, on the middle picture 12 months and on the right picture 36 months after weaning. A clear cosmetic improvement is visible when the initial surface reconstruction is compared to the last one on the right.

One explanation for the cosmetic improvement could be change of rib shape. Obviously the Rigo-Chêneau brace is able to improve cosmetic appearance by changing the shape of the thorax when the curve itself is too stiff to be corrected by a brace, while in curvatures of less than 60° with enough residual flexibility cosmetic improvements come along with improvements of the Cobb-angle (Fig. 5 and 6) [22].

Another explanation could be that a reduction of decompensation in this case leads to a better cosmetical appearance, however this would not necessarily affect ATR.

Because during development girls usually gain weight (this patient did) a third explanation could be that the bones now are more masked by fat tissue than at the start of treatment and therefore the deformation is less visible, however the patient only gained 5 kg and this would not affect the deformation in the way demonstrated here.

The rib hump seems not to be wholly a secondary effect of scoliosis as the ribs are themselves asymmetric and the hump may continue to increase, especially in immature patients even following a secure surgical spinal fusion [31]. This phenomenon is already documented in literature [32-34]. This implies that the deforming forces are still working after the operation and especially on the thoracic cage.

It is believed that in idiopathic scoliosis, the rib hump is primarily deformed because the deformity of the thorax develops first and the deformity of the central axis (the spine) succeeds [35-37].

Therefore, the conservative treatment with a brace is cutting the chain of the detrimental cascade in a critical point and it is confronting the primary problem which is the deformation of the thoracic cage, which could be straightforwardly improved. Thus a possible explanation for the clinical improvement and the radiological progression, of the scoliotic patient who is described in this paper, could be the following based on the above observations. The offered Chêneau-Brace treatment in an already rigid spine could not affect the firm central axis (the spine, which passed a threshold point for correction, being stiff and deformed) but could affect the more flexible thoracic cage correcting its deformity. In other words the thoracic cage may play the role of an intermediate buffer for the pathologically acting forces. The changing pattern of the curve as it is mentioned in the text (see Results, Patient history) could also be a triggering factor in the above alternations.

Further studies are necessary to evaluate how to predict a radiological curve progression independently from the clinical measurements, because a long lasting brace treatment with a radiological curve progression may not be recognized as a successful treatment by the patient even if the clinical improvements are obvious.

## Conclusion

Conservative treatment with out-patient exercises, in-patient rehabilitation and the Chêneau brace may improve cosmetic appearance while the curve progresses radiologically. This could be explained by assuming that (1) a well designed Chêneau brace is able to improve cosmetic appearance by changing the shape of the thorax when the curve itself is too stiff to be corrected by a brace, that (2) reduction of decompensation leads to significant cosmetical improvements or (3) that the patient gained weight and therefore the deformation is masked. However, the weight the patient gained cannot explain the cosmetical improvement.

Conservative treatment with a certain standard of quality seems a viable alternative for patients with Cobb angles of > 60° when surgical treatment is refused.

Specialists in scoliosis management should be aware of the fact that curve progression can occur even if the clinical measurements show an improvement.

## Competing interests

The author(s) declares that he has no competing interests.

## Supplementary Material

Additional File 1 History of the patient as documented by angle of trunk rotation (Scoliometer) and surface topography (lateral deviation and surface rotation) and radiological data (Cobb angle and rotation of apical vertebra). This table summarizes the data of the patient. Click here for file
